# Postoperative Knee Squeaking Following Arthroscopic Meniscal Repair: A Case‐Based Report and Literature Review

**DOI:** 10.1155/cris/3599063

**Published:** 2026-08-12

**Authors:** Amer Abdallah, Anthony Sadek, Elie Mokled, Maher Ghandour

**Affiliations:** ^1^ Faculty of Medical Science, Lebanese University, Beirut, Lebanon, ul.edu.lb; ^2^ Department of Orthopedic Surgery, Hopital Libanais Geitaoui CHU, Beirut, Lebanon; ^3^ Orthopedics Department, Aachen University Hospital, Aachen, Germany, ukaachen.de

**Keywords:** arthroscopy, conservative management, intra-articular sutures, knee squeaking, meniscal repair, postoperative complications

## Abstract

Postoperative knee squeaking is a rare phenomenon in native knees, with prior reports limited to ligament reconstructions. This case‐based report describes the first documented instances of transient knee squeaking following arthroscopic meniscal repair. We present two cases of male patients who developed audible squeaking within 1 month postoperatively using the Meniscal Cinch II (MCII) Implant Delivery System (Arthrex). Both cases demonstrated squeaking between 60° and 120° of knee motion without swelling or mechanical symptoms. Magnetic resonance imaging (MRI) findings did not reveal displaced sutures or loose bodies. Symptoms resolved spontaneously within 8 weeks without intervention. A literature review shows that previously reported cases of postoperative knee squeaking typically required arthroscopic suture removal due to intra‐articular migration. Our findings suggest that in select cases, knee squeaking may be benign and self‐limiting, supporting a conservative approach. Further studies are warranted to explore the etiology and guide management strategies for this unusual postoperative symptom.

## 1. Introduction

Postoperative squeaking in orthopedic surgery has been predominantly reported following total hip arthroplasties, particularly those utilizing ceramic‐on‐ceramic bearing surfaces [1, 2]. While this phenomenon has also been observed after total knee arthroplasty, it remains an uncommon complication [3]. A significantly rarer occurrence is the development of squeaking sounds in the native knee following arthroscopic procedures [4]. Limited literature exists regarding this postoperative complication, with only a handful of reports describing knee squeaking secondary to migrating nonabsorbable sutures. This case report presents what we believe to be the first known instance of transient knee squeaking following meniscal repair, shedding light on this rare but notable phenomenon.

## 2. Case Presentation

### 2.1. Case 1

A 37‐year‐old male with no prior medical history, allergies, or smoking habits presented with acute right knee pain following a rotational injury sustained while descending stairs. His pain, primarily localized to the medial knee and radiating posteriorly, was rated as 8/10 on the visual analog scale (VAS). He also reported sensations of popping, instability, and an inability to fully extend the knee.

Physical examination revealed joint line tenderness on the medial aspect, pain radiating posteriorly with passive flexion, and discomfort with full extension. No edema, erythema, or warmth was noted. Neurovascular examination and vital signs were unremarkable.

**Figure 1:**
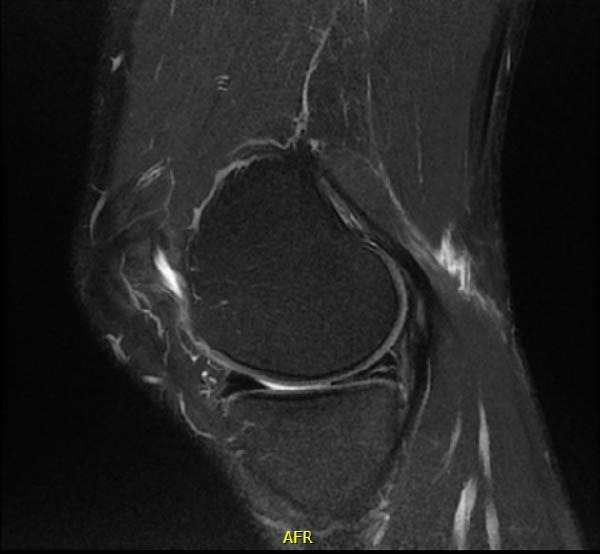
Sagittal MRI cut of right knee (Case 1) showing oblique tear in the posterior horn of medial meniscus.

Radiographs showed no acute fractures or bony lesions. Magnetic resonance imaging (MRI) confirmed an oblique tear of the posterior horn of the medial meniscus (Figure 1). Given the patient’s symptoms and imaging findings, arthroscopic meniscal repair was planned.

The surgery was performed under spinal anesthesia using two arthroscopic portals (anteromedial and anterolateral). The tear was repaired with two sutures using the Meniscal Cinch II (MCII) Implant Delivery System (Arthrex) via an all‐inside technique. The patient was discharged on postoperative day two with non‐weight‐bearing instructions for 3 weeks, restricted flexion to 90°, and a progressive physiotherapy plan.

One month postoperatively, the patient returned with complaints of a squeaking sound during knee flexion–extension, both in weight‐bearing and non‐weight‐bearing conditions. The sound occurred between 60° and 120° of knee motion and was accompanied by lateral knee pain (VAS 7/10) but no swelling (Supporting Information 1: Video 1).

**Figure 2:**
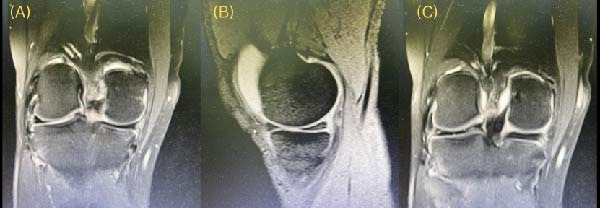
Follow‐up MRI of the right knee (Case 1) obtained 6 weeks postoperatively. (A, C) Coronal proton‐density fat‐saturated and (B) sagittal T2‐weighted images demonstrate intact healing of the previously repaired medial meniscus and a new nonarticular tear of the posterior horn of the lateral meniscus, with no loose bodies or suture migration identified.

A follow‐up MRI at 6 weeks postoperatively demonstrated a new nonarticular tear of the posterior horn of the lateral meniscus, while the previously injured medial meniscus showed intact healing signs. No loose bodies or suture migration were noted (Figure 2). By 8 weeks postoperatively, the squeaking sound had spontaneously resolved, and the patient remained asymptomatic.

### 2.2. Case 2

An 18‐year‐old male, a nonsmoker with no prior medical or surgical history, presented with severe right knee pain following a twisting injury sustained in a squatting position during MMA training. His pain was primarily lateral knee pain (VAS 9/10), associated with joint swelling, locking, and an inability to fully extend the knee.

Physical examination demonstrated tenderness over the lateral joint line, painful end‐range extension, and active extension limitation. Neurovascular examination and vital signs were within normal limits.

Radiographs revealed a mild joint effusion without fractures. MRI confirmed a complex bucket‐handle tear of the lateral meniscus, necessitating arthroscopic meniscal repair.

Surgical repair was performed using three sutures via the MCII (Arthrex) with an all‐inside technique. The patient was discharged on postoperative day two with non‐weight‐bearing instructions for 3 weeks and a progressive rehabilitation plan.

One month postoperatively, the patient reported a squeaking noise during knee motion (75°–110°), with mild discomfort (VAS 3/10) but no instability or swelling (Supporting Information 2: Video 2; Supporting Information 3: Audio). Given the absence of additional symptoms, he was advised to continue physiotherapy and conservative management. The squeaking gradually resolved after 8 weeks postoperatively without intervention.

## 3. Discussion

Squeaking in the native knee following arthroscopic procedures is an exceptionally rare phenomenon, with only a handful of documented cases in the literature. Most previous reports have described postoperative squeaking in the context of ligament reconstruction, particularly following anterior cruciate ligament (ACL), posterior cruciate ligament (PCL), and medial patellofemoral ligament (MPFL) reconstructions. However, to our knowledge, this is the first reported instance of transient knee squeaking following meniscal repair, with spontaneous resolution occurring within 8 weeks. This distinction is critical, as prior cases have almost universally required surgical intervention, primarily arthroscopic removal of migrated nonabsorbable sutures [4–6]. The findings of our study suggest that not all cases of postoperative knee squeaking necessitate immediate surgical management, and a conservative approach may be appropriate in selected cases.

A review of previously published cases reveals several important clinical characteristics associated with postoperative knee squeaking (Table 1). The time to onset varies widely, with reports documenting squeaking occurring as early as 1 month postoperatively and as late as 7 months following surgery [5]. In our cases, the squeaking was noted at approximately 1 month postoperatively, aligning with the earliest reported onset in the literature. Laterality appears to favor the right knee, as most documented cases involved the right lower extremity, except for a single case of PCL reconstruction in which the left knee was affected [5]. In terms of gender distribution, knee squeaking appears to be more common in males when occurring after ACL and PCL reconstructions [4, 5], whereas MPFL‐related squeaking has been observed exclusively in females, possibly due to differences in ligamentous laxity, anatomical factors, and surgical techniques employed in this population [6, 9].

**Table 1:** A summary of reported cases of audible squeaking post‐knee ligament reconstruction in the literature.

Study	Time to squeaking occurrence	Laterality	Gender	Associated symptoms	Investigations performed	Management	Time to resolution
Case 1 (current report)	1 month	Right	Male	Pain (VAS 7/10), no swelling	X‐ray, MRI	Conservative, resolved in 8 weeks	8 weeks (spontaneous)
Case 2 (current report)	1 month	Right	Male	Mild discomfort (VAS 3/10), no swelling	X‐ray, MRI	Conservative, resolved in 8 weeks	8 weeks (spontaneous)
Moran et al. [7]	Unknown	Unknown	Male	Psychological distress, no pain	Not specified	Surgical removal of migrating suture	Immediate after suture removal
Frazer and Talbot [8]	Postoperatively (exact time unspecified)	Right	Female	Psychological distress, no functional impact	X‐ray, MRI	Surgical debridement	Immediate after arthroscopic debridement
Izzo and Ranger [4]	2 months	Right	Female	Psychological distress, no pain	X‐ray, ultrasound, arthroscopy	Surgical removal of migrating suture	Immediate after suture removal
Amin et al. [5]	7 months	Left	Male	Pain, moderate swelling, psychosocial distress	X‐ray, MRI, arthroscopy	Surgical debridement	Immediate after debridement
Leutheuser et al. [9]	3 months	Right	Female	No pain initially, later pain with pressure	MRI, arthroscopy	Surgical removal of suture	Immediate after suture removal
Zarah et al. [6] ‐ Case 1	6 months	Right	Female	Pain, swelling, functional limitation	X‐ray, MRI, arthroscopy	Surgical removal of protruding suture	Immediate after suture removal
Zarah et al. [6] ‐ Case 2	2 months	Right	Female	No pain, only psychological distress	X‐ray, MRI, arthroscopy	Surgical removal of protruding suture	Immediate after suture removal

Abbreviations: MRI, magnetic resonance imaging; VAS, visual analog scale.

The clinical presentation of postoperative knee squeaking is highly variable. While some patients experience significant distress due to the audible nature of the phenomenon, others present with additional symptoms, including pain, swelling, and functional limitations. In cases where nonabsorbable sutures migrate into the joint space and directly contact the articular surface, patients are more likely to report pain and mechanical symptoms, whereas those without direct cartilage involvement often experience only psychological distress without functional impairment [4–6]. Our cases were characterized by the presence of transient squeaking without mechanical restriction, instability, or significant pain. The first patient reported mild discomfort (VAS 7/10), while the second described only low‐grade discomfort (VAS 3/10) that did not significantly impact the function. Notably, neither patient exhibited joint effusion, and there were no clinical or imaging findings suggestive of suture penetration into the joint.

The diagnostic workup for postoperative knee squeaking remains a challenge as conventional imaging modalities have demonstrated poor sensitivity in detecting the underlying cause. Radiographs are typically unremarkable in these cases as the sutures involved are radiolucent [4–6]. MRI has also been largely inconclusive, with multiple reports failing to identify suture migration despite clear arthroscopic confirmation [5, 6, 9]. Similarly, ultrasound has shown limited utility in detecting intra‐articular suture involvement [5]. In our cases, both MRI and radiographs failed to reveal an obvious etiology for the squeaking. This is consistent with prior findings, where arthroscopy has been the definitive diagnostic tool, often revealing migrated sutures or freely moving fixation materials that were not detected on imaging [5, 6, 9].

The precise etiology of the squeaking in our two patients remains uncertain. Because neither radiographs nor MRI demonstrated suture migration, loose bodies, or articular impingement, and because both episodes resolved spontaneously, we postulate that the sound arose from transient friction within the early postoperative construct rather than from mechanical derangement. Plausible sources include friction between the high‐strength nonabsorbable suture and the polymer (PEEK) backstop implants of the all‐inside delivery system, friction between adjacent suture bridges, or friction between the construct and the surrounding synovium and soft tissues before implant settling and tissue remodeling occurred. The reproducibility of the sound within a discrete mid‐flexion arc (60°–120°) is consistent with a localized frictional interface that is loaded only through part of the range of motion. Notably, both repairs were performed with the same all‐inside system (MCII, Arthrex), raising the possibility of a device‐ or technique‐related contribution; for example, backstop implants or suture limbs positioned in close proximity could transiently rub against one another or against the joint capsule. The two procedures were performed in June 2024 and March 2025; at that time, the operating surgeon had been using the MCII system since 2016 (approximately eight to 9 years of experience). We emphasize; however, that with only two cases and without intraoperative or arthroscopic confirmation of the responsible interface, a causal relationship to the implant system cannot be established, and these mechanisms remain hypothetical. The spontaneous resolution observed in both patients argues against suture cut‐through or intra‐articular migration, which in previously reported cases has typically required arthroscopic removal.

Management strategies for postoperative knee squeaking depend on the severity of the symptoms and the presence of functional impairment. In most published cases, surgical intervention was required, particularly when imaging or clinical findings suggested intra‐articular suture migration [4–6]. Diagnostic arthroscopy with suture removal or debridement has been the preferred approach, and in all surgically treated cases, the squeaking resolved immediately following the procedure. However, our cases differed in that the squeaking was self‐limiting and resolved spontaneously within 8 weeks. This suggests that not all instances of postoperative knee squeaking necessitate immediate surgical intervention, particularly in cases where the symptoms are mild and there is no evidence of direct cartilage impingement.

Given these findings, a stepwise approach to management is warranted. Patients presenting with postoperative knee squeaking should undergo a thorough clinical evaluation to assess for signs of instability, mechanical restriction, or pain. If symptoms are minimal and imaging does not reveal significant abnormalities, a trial of conservative management with observation and structured physiotherapy may be reasonable. In cases where symptoms persist beyond 8–12 weeks or are associated with significant discomfort or functional limitation, diagnostic arthroscopy should be considered [5, 6]. Surgical intervention is particularly indicated in cases where suture migration is suspected or if there is evidence of persistent friction between the suture and the articular cartilage as spontaneous resolution in these cases appears unlikely [4–6].

This report presents the first documented cases of transient knee squeaking following meniscal repair that resolved spontaneously without surgical intervention. While previous reports have largely emphasized the need for arthroscopic debridement or suture removal, our findings highlight the potential for a conservative approach in select cases. Further studies are needed to better understand the pathophysiology of this phenomenon, identify risk factors for persistent symptoms, and establish guidelines for determining when surgical intervention is necessary. Until then, clinicians should carefully evaluate each case on an individual basis, balancing the risks of unnecessary surgery against the potential benefits of early arthroscopic intervention in cases where spontaneous resolution is unlikely.

## Acknowledgments

The authors have nothing to report.

## Funding

This research did not receive any specific grant from funding agencies in the public, commercial, or not‐for‐profit sectors.

## Consent

Written informed consent was obtained from the patient for the publication of anonymized clinical details included in the manuscript.

## Conflicts of Interest

The authors declare no conflicts of interest.

## Supporting Information

Additional supporting information can be found online in the Supporting Information section.

## Supporting information


**Supporting Information 1** Video 1 (Case 1): Video of Case 1 demonstrating the audible knee squeaking during active flexion–extension following arthroscopic meniscal repair. On‐screen labels mark the moments at which the squeak is heard, recurring with each flexion–extension cycle (first clearly audible at approximately 1 s into the clip).

**Supporting Information 2** Video 2 (Case 2): Video of Case 2 demonstrating knee motion following arthroscopic meniscal repair.

**Supporting Information 3** Audio (Case 2): Audio recording of the knee squeaking in Case 2. The squeak is captured as discrete high‐pitched events recurring with knee motion (audible, for example, at ~4, 5.5, 7.5, and 12 s).

## Data Availability

The data used to support the findings of this study are available from the corresponding author upon request.
